# Neural Causal Information Extractor for Unobserved Causes

**DOI:** 10.3390/e26010046

**Published:** 2023-12-31

**Authors:** Keng-Hou Leong, Yuxuan Xiu, Bokui Chen, Wai Kin (Victor) Chan

**Affiliations:** 1 Tsinghua Shenzhen International Graduate School, Tsinghua University, Shenzhen 518055, China; liangjh22@mails.tsinghua.edu.cn (K.-H.L.); xiuyx19@mails.tsinghua.edu.cn (Y.X.); 2 Tsinghua-Berkeley Shenzhen Institute, Tsinghua University, Shenzhen 518055, China; 3 Peng Cheng Laboratory, Shenzhen 518055, China; 4 International Science and Technology Information Center, Shenzhen 518055, China

**Keywords:** causal inference, maximizing mutual information, unobserved causes, complex system

## Abstract

Causal inference aims to faithfully depict the causal relationships between given variables. However, in many practical systems, variables are often partially observed, and some unobserved variables could carry significant information and induce causal effects on a target. Identifying these unobserved causes remains a challenge, and existing works have not considered extracting the unobserved causes while retaining the causes that have already been observed and included. In this work, we aim to construct the implicit variables with a generator–discriminator framework named the Neural Causal Information Extractor (NCIE), which can complement the information of unobserved causes and thus provide a complete set of causes with both observed causes and the representations of unobserved causes. By maximizing the mutual information between the targets and the union of observed causes and implicit variables, the implicit variables we generate could complement the information that the unobserved causes should have provided. The synthetic experiments show that the implicit variables preserve the information and dynamics of the unobserved causes. In addition, extensive real-world time series prediction tasks show improved precision after introducing implicit variables, thus indicating their causality to the targets.

## 1. Introduction

**Figure 1:**
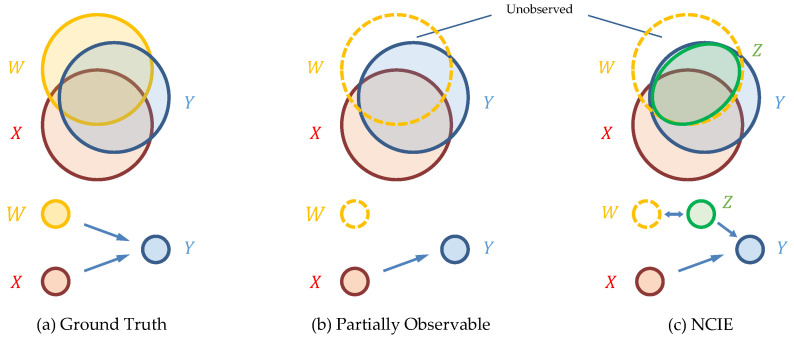
Illustration of the information in a Venn diagram, as well as the causal relationships of the (**a**) ground truth; (**b**) partially observable scenario in reality; and (**c**) an alternative representation of causal relationships with the implicit variables *Z*, which is generated by NCIE, where *Y*, *X*, and *W* denote the target, observed causes, and unobserved causes, respectively.

Identifying the complete and valid causes of a variable has been a significant pursuit in causal inference toward complex systems. A causal inference approach is called “causally faithful” [1] when it reveals the causal relationships shown in the observed variables, and it is “causally sufficient” [2] if it does not omit any of the hidden confounder or unobserved variables that should be included. To hold causal sufficiency when deriving faithful causal relationships, traditional causality inference methods [1,2,3,4,5,6] often assume the complete observability of the system, i.e., all of the variables and information within the system are collected and observed. In the perspective of [7,8], the information within the system flows from the causes to the targets; thus, the complete causes can provide most of the target’s information and can describe it (except the noise terms) in detail, as shown in Figure 1a.

However, in practical scenarios, complex systems are often partially observable since only a subset of variables can be directly measured. For instance, in financial markets, the influence of media [9] and company-specific factors [10], which are hardly measurable, drive important causal effects in local tradings [9] and price reversals [10]. Another example can be shown in ecosystems, in which the dynamics are described by species populations, habitat quality, and threats; however, one can rarely acquire a perfect knowledge of the system states [11,12] to perform adequate management actions. The partial observability also induces analyzing challenges in domains such as monitoring biophysical objects [13], weather and climate forecasting [14], and industrial management [15]. While part of the causes are unobserved, the target’s information that should be provided is unknown, as shown in Figure 1b. To describe the causal structures in such incomplete systems, causal sufficiency in traditional works is no longer guaranteed, and their results do not necessarily identify the real causes even when they are causally faithful [1,16].

Therefore, to avoid identifying spurious causes, the common idea shared by most practices [17,18,19,20,21,22,23,24,25,26,27,28] is to introduce implicit variables that compensate for the missing causes. Most of them have applied neural networks as a powerful tool for generating variables, which could be categorized into two streams: either (i) employing neural networks with latent layers to uncover the significant features in observed variables that better formulate the targets, or (ii) utilizing the ability to preserve information in representation learning to extract unobserved or latent causes that provide significant information of the targets.

The works from the first approach adopt observed causes as the inputs to train a neural network that minimizes the fitting loss between the output and the target. The intermediate layers in the neural networks help to better fit the target in supervised learning [17], for example, by capturing the hidden long-/short-term dependencies in the dynamics [18,19]. Granger [29] suggested that we could consider these hidden variables as causes, since including them could perform a better regression of the targets. However, such methods are, in actuality, just recombining the neurons from the input to extract advanced features, and they cannot provide information beyond the input [30]. Therefore, they do not necessarily provide unobserved information; thus, we can hardly identify these hidden variables as those that are unobserved in the system.

The second stream aims to generate implicit variables that provide more information to the targets. This echoes another definition of causality [3,31], in which a cause should conditionally correlate with the target given any of the sets of other variables, or it could provide information that all the other variables could not provide to the target [32]. These methods [20,21,22,23,24,25,26,27,28] adopt the idea of representation learning, which inputs the target (and all the observed variables) into neural networks to project an embedding. This preserves the information while describing the prominent dynamics of the original data. Since these latent variables generate a cover of most of the targets’ information, they could be referred to as the causes that drive the system. However, in general, the observed and unobserved causes are entangled and inseparable in the causal structure. While refs. [20,21,22,23,24,25,26,27,28] excluded all of the observed causes and only considered the generated latent variables as the targets’ causes, their depiction does not necessarily reveal the real causal relationships.

Motivated by the aforementioned issues of either missing the unobserved information or giving up on the observed causes, this paper proposes a framework to complete the causal structure by extracting the unobserved variables while retaining the observed causes. We assume the existence of unobserved causes to the target variables; hence, the targets’ information should be provided by both the observed and unobserved causes. Given the part of the information from the observed causes, our objective is to generate implicit variables from the observed variables that provide as much information to the target as possible, thus covering the information that should have been provided by the unobserved causes. As shown in Figure 1c, the implicit variables *Z* would cover the information of the target *Y* that should have been provided by the unobserved causes *W*, and hence it could provide an alternative representation ( {X,Z}→Y ) of the information flow in the causal relationships {X,W}→Y .

In detail, we employ a generator–discriminator architecture named the Neural Causal Information Extractor (NCIE) that extracts and embeds the targets’ information into the implicit variables. The generator obtains implicit variables fromthe time series inputs, and the discriminator maximizes the mutual information between the targets and the union of explicit and implicit causes by constraining implicit variables to have diverse marginal and joint distributions. This results in a holistic causal structure that encompasses both observed and unobserved factors to the target. To highlight the ability of NCIE to complement unobserved information and recover unobserved dynamics, we generate implicit variables for three synthetic cases. To further demonstrate the efficacy of the generated implicit causes in improving time series predictions and to verify their causality, we perform extensive experiments on real-world time series data.

The contribution of our work can be highlighted as follows:

1. We propose a generator–discriminator architecture that generates implicit variables to complement the unobserved causes in the causal structures while retaining the observed causes.
2. The implicit variables we generate could carry information from the unobserved variables and reveal their dynamics.
3. Time series prediction tasks show that the combination of observed and implicit variables helps improve the prediction of targets, verifying that they are better candidates for causal inference.

This paper is organized as follows. Section 2 provides a comprehensive review of related works. Section 3 presents the basic theories in information theory and causal inference and states the objective of this work. Section 4 introduces the methodology of the proposed approach. Section 5 details the experimental setup and dataset descriptions, and then presents the results and discussion of our experiments. Finally, in Section 6, we provide a discussion on the implications of our work and present future possible advanced works.

## 2. Literature Review

In the scope of our work, we categorize the related works on causal inference into two parts, which cover the methods with and without considering unobserved variables.

### 2.1. Causal Inference without Considering Unobserved Variables

Assuming that the given observations are complete and thus sufficient, the objectives of traditional works often include drawing causal faithful graphs, in which nodes represent the variables of interest and edges indicate the existence of causal relationships between variables. The constrained-based methods, including the Peter–Clark (PC) algorithm [2] and its improvements [2,3,33], are graph algorithms that start with a fully connected graph and iteratively delete the edges between two variables if they are conditionally independent or d-separated [2]. They provide causal faithful graphs that satisfy the causal Markov condition [31], i.e., a variable is conditionally independent to all variables except its effects given its causes. On the other hand, score-based approaches present the causal structure with the Bayesian network with the highest score, measured by the Bayesian Dirichlet equivalent (BDe) score [4] and the K2 score [5]. Though the latter methods do not ensure faithfulness, they pursue a representation that shows the causal structure and, at the same time, the strength of cause-to-effect dependencies. Other approaches include those based on Granger causality [6], which also aim to identify the existence and magnitudes of the causal edges among variables.

### 2.2. Causal Inference Considering Unobserved Variables

There are plenty of works on causal inference that are aware of the existence of unobserved variables, and for clarity, we attempt to classify them into three groups. The first group refers to those that detect the existence of hidden confounders without knowing the effects, including Fast Casual Inference (FCI) [2] and Latent PCMCI (LPCMCI, where PCMCI [3] is a PC-based condition selection with the Momentary Conditional Independence (MCI) test) [34]. They are the modified versions of the PC algorithm [2] and PCMCI [3], respectively, which follow similar procedures but identify an extra kind of edge (relationship) between variables: bidirectional arrows. This indicates the conditional correlational relationship between two variables that could not be explained by any directional causal path from one to another, thus identifying the existence of a hidden confounder driving the two variables.

The second group attempts to explain the causal effects originating from unobserved variables toward the target variable. Assuming linear, additive, and first-order Markovian causal effects, ref. [16] constructs a linear regression model that describes the observed variables by past observed variables, unobserved causes, and noises. Based on this model, the effects of the unobserved causes, which refer to their coefficients, could be obtained by variational expectation maximization or by solving the autocovariance.

The third group [20,21,22,23,24,25,26,27,28] aims to identify the unobserved causes via latent representations. They assume that the observed signals are driven or caused by a set of unobserved latent signals, and there exists a latent dynamic that drives the latent variables from past to future. Therefore, given the observed dynamics, their objectives are to identify the posterior distributions of future latent signals given past latent signals, the posterior distributions of observed signals given the contemporaneous latent signals, and the latent signals themselves. They identify latent signals via different approaches, including Variational Autoencoder (VAE) [20,21,35,36], Dynamical Component Analysis [25,26,27], and maximizing the (conditional) mutual information [22,28].

While refs. [20,21,22,23,24,25,26,27,28] ensure the identifiability of implicit variables, their structures do not include the causes that are already observed, which might also be involved in the causal process. In our work, we express the causal structures containing both observed and implicit causes. Moreover, while refs. [20,21,22,23,24,25,26,27,28] could precisely identify the implicit variables, many of their generators, such as VAE [20,21,35,36], hold specific assumptions about the implicit variables. With a simple generator that holds comparably few assumptions about the distribution, our architecture can be applied to more general cases.

### 2.3. Mutual Information Estimator


(1) $$\begin{array}{c} H\left(Y\right)=-\sum_{y\in Y}p\left(y\right)log\left(p\left(y\right)\right),\\ H\left(Y\right)=-\int_{Y}p\left(y\right)log\left(p\left(y\right)\right)dy. \end{array}$$

In Equation (1), Shannon provided an information theory-based definition for the entropy of random variables with known distributions. However, the distribution of variables in real-world time series remains unknown in many scenarios. To sample the unknown distributions, one of the fundamental methods is binning [37,38]. By partitioning the continuous variables into discrete bins and counting the sampled distributions, one can apply Equation (1) to estimate entropy. Provided the entropy of variables, one can calculate the mutual information between two variables, *X* and *Y*, with

(2) $$\begin{array}{cc} I(X;Y) & \;=\sum_{x\in X}\sum_{y\in Y}p_{XY}\left(x,y\right)\log\left(\frac{p_{XY}\left(x,y\right)}{p_{X}\left(x\right)p_{Y}\left(y\right)}\right)\\ \; & \;=H(X)+H(Y)-H(X,Y). \end{array}$$

The Mutual Information Neural Estimator (MINE) [39] is another popular method that uses a neural network to estimate the KL Divergence of two random variables, which could then provide a tight lower bound for their mutual information. Deep InfoMax [22] provides a similar approach by estimating Jensen–Shannon (JS) Divergence. Under the Gaussian assumption, Refs. [25,26] measure entropy by $H\left(Z\right)=\frac{r1}{2}ln{\left(2\pi e\right)}^{dT}\left|\Sigma_{T}\left(Z\right)\right|$ , and prove that the estimation is still tight if the assumption is violated. Refs. [40,41,42] provide other effective estimators for information based on classifiers and k-nearest neighbor (kNN).

## 3. Problem Statement

Suppose we have target variable $Y_{t}$ at time *t*. First, we consider the case where $Y_{t}$ is auto-correlated, and all the causes of $Y_{t}$ are known, denoted by $\left\{X_{t},Y_{<t}\right\}$ , where $X_{t}={\left\{X^{1},\;X^{2},\;...\;,X^{n}\right\}}_{t}$ and $Y_{<t}=\left\{Y_{1},\;...\;,\;Y_{t-1}\right\}$ . In this work, we assume a *k*-order Markovian property on auto-correlated causality, i.e., $Y_{<t}=\left\{Y_{t-k},\;...\;,\;Y_{t-1}\right\}$ .

For simplicity, we abbreviate $Y_{t}$ as *Y*. While all the causes $\left\{X,Y_{<t}\right\}$ are known, we assume

(3) $$H\left(Y\right)=I\left(Y;\;X,\;Y_{<t}\right),$$

where H(Y) is the entropy of *Y*, a measure of uncertainty, and $I(Y;\;X,\;Y_{<t})$ is the mutual information (MI) between *Y* and $\left\{X,\;Y_{<t}\right\}$ . MI is a measure of “information” or “reduction of entropy” that the two variables provide to each other. In Equation (3), the entropy reduction that *X* and $Y_{<t}$ provide to *Y* equals the entropy of *Y*. Hence, the causes of *Y* could eliminate all the uncertainty of *Y*, in other words, provide all the information of *Y*.

Now, we consider another case in which, besides some observed causes $X=\{X^{1},\;X^{2},\;$ $...\;,\;X^{n}\}$ , there are other unobserved causes *W*. Therefore, we assume

(4) $$H\left(Y\right)=I\left(Y;\;X,\;Y_{<t},\;W\right).$$

The absence of information about *W* induces

(5) $$H\left(Y\right)>I\left(Y;\;X,\;Y_{<t}\right).$$

**Figure 2:**
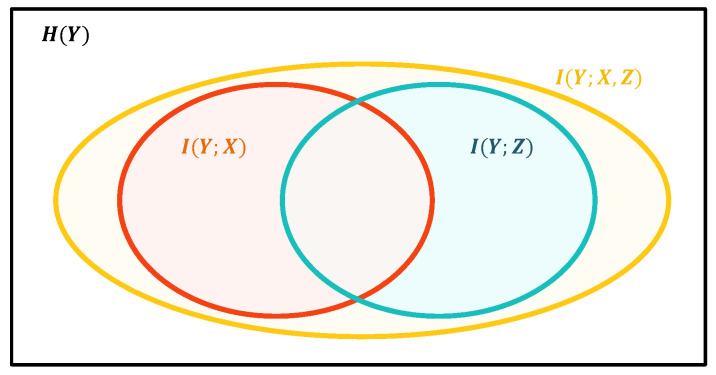
The Venn Diagram illustration of the information provided to *Y* by *X* (red), *Z* (blue), and {X,Z} (yellow).

As shown in Figure 2, *X* and $Y_{<t}$ could only provide a part (the red part) of the information to *Y*. Therefore, our objective is to generate some implicit variables $Z=\{Z^{1},\;Z^{2},\;...\;,\;Z^{m}\}$ to maximize $I(Y;\;X,\;Y_{<t},\;Z)$ (the yellow part), which attempts to approach its upper bound H(Y) (the large black box).

Hence, if we could search for a *Z* that could perfectly provide this information, we could say that *Z* somehow represents the unobserved causes *W*. More specifically, *Z* traces the information for *Y* from *W*.

While the upper bound of the MI equals the entropy of *Y*, i.e.,

(6) $$\underset{Z}{\sup}I\left(Y;\;X,\;Y_{<t},\;Z\right)=H\left(Y\right),$$

the objective of our work is to generate *Z* which maximizes the MI and makes it approach H(Y) :

(7) $$\max_{Z}I\left(Y;\;X,\;Y_{<t},\;Z\right),$$

which is equivalent to maximizing the conditional mutual information of *Z* and *Y* given *X* and $Y_{<t}$ :

(8) $$I\left(Y;Z|X,\;Y_{<t}\right)=I\left(Y;\;X,\;Y_{<t},\;Z\right)-I\left(Y;\;X,\;Y_{<t}\right).$$

Since the term without *Z* could be considered constant here,

(9) $$\max_{Z}I\left(Y;\;X,\;Y_{<t},\;Z\right)=\max_{Z}I\left(Y;Z|X,\;Y_{<t}\right).$$

Objective (7) is maximized if *Z* covers all the conditional mutual information from *W*,

(10) $$\underset{Z}{\sup}I\left(Y;\;Z|\;X,\;Y_{<t}\right)=I\left(Y;\;W|\;X,\;Y_{<t}\right)=H\left(Y|X\right),$$

and meanwhile $X,Y_{<t},Z$ together cover all the entropy (Equation (6)) and provide all the information of *Y*.

There are some assumptions made on *Z*:

1. We focus on the unobserved variables that provide information to *Y*. Those without information of *Y* do not affect the distributions of *Y*.
2. In the case of time series, we denote $X_{<t}$ , $Z_{<t}$ as the explicit and implicit causes of $Y_{t}$ to uphold the assumption of temporal priority [1], i.e., causal relationships only move from past variables to future variables in time series. For simplicity, we denote $X_{<t}$ and $Z_{<t}$ as *X* and *Z*, and $Y_{t}$ as *Y*.
3. We assume $X_{t}$ , $Y_{t}$ , and $Z_{t}$ are in a closed system and there is a causal loop among them, i.e., $X_{<t}$ and $Z_{<t}$ may induce $Y_{t}$ , and $X_{<t}$ and $Y_{<t}$ may induce $Z_{t}$ .
4. While we are generating $Z_{<t}$ , we do not necessarily obey temporal priority [1], which is a common practice in refs. [20,21,22,23,24,25,26,27,28,30]. Therefore, we employ $X_{<t}$ and $Y_{t}$ to generate $Z_{<t}$ . We note that we do not focus on finding the exact causes of *Z* here. Instead of finding causes of *Z* by applying the whole structures we use on *Y*, we just use a basic RNN to present *Z* from the explicit causes.

To better illustrate our problem and objective, we provide a simple numerical example as follows:

**Example** **1.** *We consider a temporal Boolean network with three Boolean variables: $Y_{t}$ , $X_{t}$ , and $W_{t}$ , where $Y_{t}$ at time t is determined by*

(11) $$Y_{t}=X_{t-1}\;\mathrm{AND}\;W_{t-1}.$$

*At every time step t, $X_{t}$ and $W_{t}$ are sampled from a uniform binomial distribution, i.e., $P\left(X_{t}=0\right)=P\left(X_{t}=1\right)=P\left(W_{t}=0\right)=P\left(W_{t}=1\right)=0.5.$
*

*In this context, the time-varying variables $Y_{t}$ , $X_{t-1}$ , and $W_{t-1}$ at a particular time t are considered as the static random variables generated by the random process [1,2,3,22,24,28]. We list all the possible circumstances of the truth table of these three variables.*

(12) $$\begin{array}{c} P(X_{t-1}=0,W_{t-1}=0,Y_{t}=0)=1/4;\\ P(X_{t-1}=0,W_{t-1}=1,Y_{t}=0)=1/4;\\ P(X_{t-1}=1,W_{t-1}=0,Y_{t}=0)=1/4;\\ P(X_{t-1}=1,W_{t-1}=1,Y_{t}=1)=1/4. \end{array}$$

*Then, we calculate $H(Y_{t})$ , $I(Y_{t};X_{t-1})$ , and $I(Y_{t};X_{t-1},W_{t-1})$ respectively:*

(13) $$\begin{array}{cc} H\left(Y_{t}\right)=-\sum_{y\in Y_{t}} & p\left(y\right)\log\left(p\left(y\right)\right)=2-\frac{3}{4}log3,\\ I\left(Y_{t};X_{t-1}\right)\;=\sum_{y\in Y_{t}}\sum_{x\in X_{t-1}} & p\left(x,y\right)\log\left(\frac{p(x,y)}{p(x)p(y)}\right)=\frac{3}{2}-\frac{3}{4}\log3,\\ I\left(Y_{t};X_{t-1},W_{t-1}\right)\;=\sum_{y\in Y_{t}}\sum_{x\in X_{t-1}}\sum_{w\in W_{t-1}} & p\left(w,x,y\right)\log\left(\frac{p(w,x,y)}{p(w,x)p(y)}\right)=2-\frac{3}{4}\log3. \end{array}$$

*We notice that $H\left(Y_{t}\right)=I\left(Y_{t};X_{t-1},W_{t-1}\right)>I\left(Y_{t};X_{t-1}\right)$ , which means that the information of $Y_{t}$ could be completely covered when $X_{t-1},W_{t-1}$ are known. When $W_{t-1}$ is unobserved, the observed $X_{t-1}$ can only provide part of the information of $Y_{t}$ . This gap of information is described by the conditional mutual information, $I(Y_{t};W_{t-1}|X_{t-1})$ , where*

(14) $$\begin{array}{c} I\left(Y_{t};W_{t-1}|X_{t-1}\right)=I\left(Y_{t};X_{t-1},W_{t-1}\right)\;-I\left(Y_{t};X_{t-1}\right)=\frac{1}{2}, \end{array}$$

*which quantifies the information that $W_{t-1}$ provides to $Y_{t}$ that $X_{t-1}$ cannot provide.*

**Example** **2.** *Now, we consider the scenario in which we do not know $Y_{t}$ is generated by Equation (11) or the distribution of $W_{t-1}$ , but we are provided with $X_{t-1}$ and $Y_{t}$ , and we wish to generate $Z_{t-1}$ to complement $I(W_{t-1};Y_{t}|X_{t-1})$ and maximize the objective in Equation (7). The ideal case is to reverse the Markov chain ( $W_{t-1},X_{t-1}\to Y_{t}$ ) to ( $X_{t-1},Y_{t}\to W_{t-1}$ ) and make the posterior distribution $p(Z_{t-1}|X_{t-1},Y_{t})$ equal to $p(W_{t-1}|X_{t-1},Y_{t})$ , where the joint and posterior distributions are*

(15) $$\begin{array}{c} P(X_{t-1}=0,Y_{t}=0,Z_{t-1}=0)=1/4,\\ P(X_{t-1}=0,Y_{t}=0,Z_{t-1}=1)=1/4,\\ P(X_{t-1}=1,Y_{t}=0,Z_{t-1}=1)=1/4,\\ P(X_{t-1}=1,Y_{t}=1,Z_{t-1}=0)=1/4, \end{array}$$

(16) $$\begin{array}{c} p\left(Z_{t-1}=1|X_{t-1},Y_{t}\right)=p\left(W_{t-1}=1|X_{t-1},Y_{t}\right)=\left\{\begin{array}{cc} 1, & X_{t-1}=1,Y_{t}=1\\ 0, & X_{t-1}=1,Y_{t}=0\\ \frac{1}{2}, & X_{t-1}=0 \end{array}\right.. \end{array}$$

*In such a case, we calculate that*

(17) $$\begin{array}{cc} \; & I\left(Y_{t};X_{t-1},Z_{t-1}\right)=I\left(Y_{t};X_{t-1},W_{t-1}\right)=2-\frac{3}{4}log3,\\ \; & I\left(Y_{t};Z_{t-1}|X_{t-1}\right)=I\left(Y_{t};W_{t-1}|X_{t-1}\right)=\frac{1}{2}. \end{array}$$

**Example** **3.** *However, there actually exist more than one $p(Z_{t-1}|X_{t-1},Y_{t})$ that can maximize $I(Y_{t};Z_{t-1}|X_{t-1})$ . For example, we let*

(18) $$Z_{t-1}=X_{t-1}\;\mathrm{XOR}\;Y_{t},$$

*and we can again list all the possible circumstances of the truth table of these three variables.*

(19) $$\begin{array}{c} P(X_{t-1}=0,Y_{t}=0,Z_{t-1}=0)=1/2,\\ P(X_{t-1}=1,Y_{t}=0,Z_{t-1}=1)=1/4,\\ P(X_{t-1}=1,Y_{t}=1,Z_{t-1}=0)=1/4. \end{array}$$

*In such a case, we can obtain the same result as that in Equation (17), which means a different Z from Equations (16) and (18) can achieve the optimal objective in (7). This indicates that they are providing the same conditional information that W provides to Y. The value of $Y_{t}$ is known when $X_{t-1}=0$ , but is uncertain when $X_{t-1}=1$ . W provides the conditional information by helping to eliminate this uncertainty (or conditional entropy H(Y|X) ), which can also be eliminated by not including W but including Z from Equation (16) or (18). Therefore, Z can express conditional information I(W;Y|X) .*

## 4. Methods

### 4.1. Neural Causal Information Extractor

**Figure 3:**
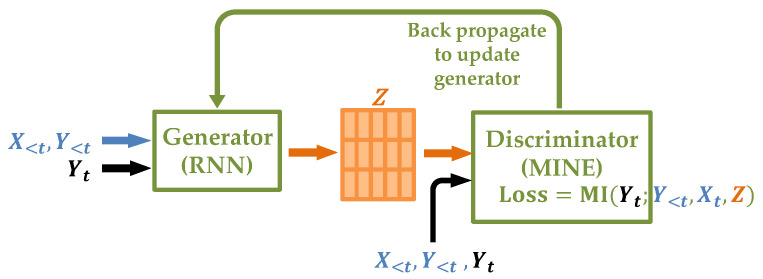
The architecture of the Neural Causal Information Extractor.

We name our architecture the Neural Causal Information Extractor (NCIE), which is composed of a generator and a discriminator. The architecture is shown in Figure 3.

#### 4.1.1. Generator

Although *Z* is not explicitly collected and observed from the system, we consider part of it to be involved in the system, specifically the part that provides information to *Y*. While we suppose that *Y* is generated by $\left\{X,Y_{<t},Z\right\}$ , reversely, the information of *Z* that we are interested in should be covered by (a part of) *Y*. Therefore, *Z* could be represented as a function of *X*, $Y_{<t}$ , and *Y*. Other variables *V* besides $\left\{X,Y_{<t},Y\right\}$ in the raw data should not be included to generate *Z* in case they have not provided information via path V→Z→Y (or *V* should be detected as an observed direct cause before *Z* is included).

While finding the causes of *Z* is not the focus of this work (we only focus on finding the explicit and implicit causes of $Y_{t}$ ), we apply a basic RNN [18] to capture the lagged dependencies from *X* and *Y* to generate *Z*. The RNN consists of two neural networks to manipulate and propagate its hidden states. The first neural network, $f_{1}$ , generates $H_{t}$ from $X_{<t}$ , $Y_{<t}$ , $Y_{t}$ , and $H_{t-1}$ (Equation (20)), where $H_{t}$ denotes the hidden states at time *t*. Then, the second neural network, $f_{2}$ , reads $H_{t}$ and generates the output *Z* (Equation (21)), while $H_{t}$ is propagated to the next episode.

(20) $$\begin{array}{cc} H_{t}= & f_{1}\left(X_{t-1},\;Y_{t-1},\;H_{t-1}\right), \end{array}$$

(21) $$\begin{array}{cc} Z_{t}= & f_{2}\left(H_{t}\right). \end{array}$$

With the propagation of $H_{t}$ , the memory of *X* and *Y* in previous episodes is also propagated. Furthermore, since *Z* is generated directly from *H*, the propagation of *H* also passes the memory and information of *Z*. It is worth noting here that *Z* is jointly generated by the explicit {X,Y} and the implicit variables $Z_{<t}$ . Suppose we employ another architecture that generates *Z* solely by explicit variables {X,Y} (and without $Z_{<t}$ ); *Z* then acts as an intermediate hidden layer in our architecture, which is equivalent to directly depicting *Y* by a neural network structure with {X,Y} and without *Z*. In other words, *Y* would then be solely caused by explicit variables. This is why we apply RNN to generate *Z* jointly by implicits and explicits, which matches our assumption that *Y* is causally related to implicits and explicits.

#### 4.1.2. Discriminator

The discriminator provides a measurement of $I(Y;X,Y_{<t},Z)$ , where *Z* is the output of the generator. Here, we consider *Y* and $\left\{X,Y_{<t},Z\right\}$ as two sets of random variables, and their mutual information is equivalent to Kullback–Leibler (KL) divergence between their marginal and joint distributions,

(22) $$\begin{array}{cc} I(Y;X,Y_{<t},Z)\;\; & =\;D_{KL}(P_{YXY_{<t}Z}\;||\;P_{Y}⨂P_{XY_{<t}Z})\\ \; & =\;\underset{T:Y\times X\times Y_{<t}\times Z\to R}{\sup}E_{P_{YXY_{<t}Z}}\left[T\right]-\log\left(E_{P_{Y}⨂P_{XY_{<t}Z}}\left[e^{T}\right]\right) \end{array},$$

where $D_{KL}$ denotes the KL divergence of two distributions and $P_{Y}$ denotes the distribution of *Y*. The lower bound of KL divergence can be provided by Donsker–Varadhan representation (Equation (22)), where $T(Y,X,Y_{<t},Z)$ is any class of function.

We follow MINE [39] to implement function *T* by a neural network with parameters Θ and maximize the lower bound of mutual information $I_{\Theta }$ by searching for optimized function $T_{\theta }$ :

(23) $$I\left(Y;X,Y_{<t},Z\right)\geq I_{\Theta }\left(Y;X,Y_{<t},Z\right),$$

(24) $$I_{\theta }\left(Y;X,Y_{<t},Z\right)=\max_{\theta \in \Theta }E_{P_{YXY_{<t}Z}}\left[T_{\theta }\right]-\log\left(E_{P_{Y}⨂P_{XY_{<t}Z}}\left[e^{T_{\theta }}\right]\right).$$

Optimization is performed using stochastic gradient descent (SGD). However, a naive derivation of Equation (24) leads to biased gradient estimation. MINE [39] proposed addressing this bias by reformulating the loss function. For batch sample *B*, the gradient is

(25) $$\hat{G_{B}}=E_{B}\left[\nabla_{\theta }T_{\theta }\right]-\frac{E_{B}\left[\nabla_{\theta }T_{\theta }e^{T_{\theta }}\right]}{E_{B}\left[e^{T_{\theta }}\right]}.$$

MINE suggests estimating the denominator of the second term using the moving average from previous epochs, while the other parts are the averaged gradient from the network parameters.

Among all (conditional) mutual information estimators, MINE estimates MI by stochastic gradient descent (some do not provide MI, and some are not trained by gradient descent). The available gradient is essential in our architecture because one of the inputs of MINE, *Z*, is exactly the output of the generator. Hence, the generator and discriminator are fused together by *Z*, which acts as the bridge to back-propagate the gradient from the discriminator to the generator and thus update the parameters in both neural networks.

#### 4.1.3. NCIE: Maximizing Mutual Information

In Section 3, we claim that the objective of our architecture is to find implicit variables *Z* such that $I(Y;X,Y_{<t},Z)$ is maximized. In this case, *Z* could represent the information provided by unobserved variables.

Since the generator and discriminator are fused together, they could be jointly considered as a whole neural network that trains both components simultaneously, where *Z* could be viewed as a layer in between that we are interested in. While training together by gradient descent, the generator is trained to provide better *Z* to push up $I_{\theta }$ , and the discriminator is trained to provide a better (higher) estimation of $I_{\theta }$ given the *Z* from the generator. Therefore, NCIE pursues objective

(26) $$\max_{Z,\;\theta \;\in \;\Theta }I_{\theta }\left(Y;X,Y_{<t},Z\right).$$

In an alternative view, the whole NCIE structure could be interpreted as a variant of an RNN that pursues to provide a maximized value of the Donsker–Varadhan representation given the batched inputs of *X*, $Y_{<t}$ , and *Y*.

### 4.2. Verifying Causality from Z to Y by Time Series Prediction

#### 4.2.1. Motivation and Architecture

To verify the capability of extracting effective implicit causes, we modify the NCIE in Section 4.1 and apply the architecture to perform multi-variate time series prediction in Section 5.2.2. To predict $Y_{t}$ , we cannot use $Y_{t}$ itself or any variables at time *t* or after *t* as the input; therefore, we have to find an alternative for the input of the generator. Here, we use a simple RNN to pre-train a $\hat{Y_{t}}$ that tries to estimate $Y_{t}$ given all the explicit causes $\left\{X,Y_{<t}\right\}$ of $Y_{t}$ . After the pre-training stage, $\hat{Y_{t}}$ is plugged into the input of the generator in the NCIE and replaces $Y_{t}$ , to avoid the information leakage in the original architecture in Section 4.1.

**Figure 4:**
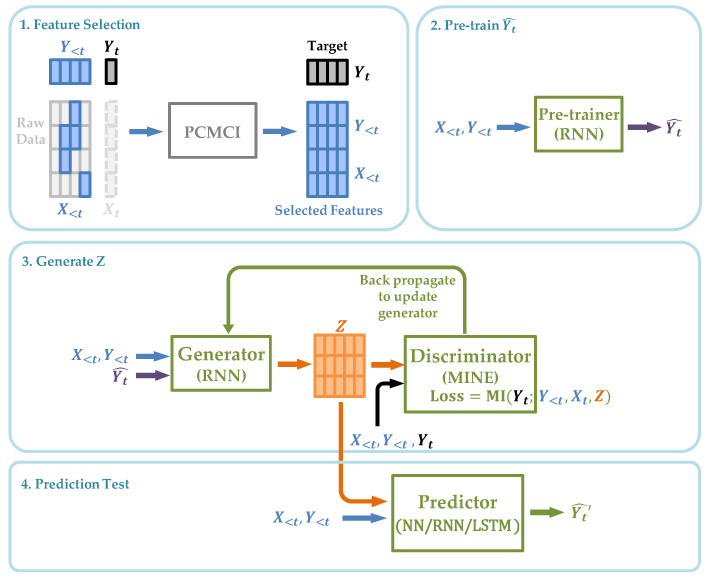
The architecture of Neural Causal Information Extractor to perform time series prediction.

The overall procedure is shown in Figure 4, which is summarized as follows:

1. We select the explicit causes *X* from the raw time series with a maximum lag of *k* using PCMCI [3], which is a framework that identifies the time-lagged causal relationship given the assumption of the completeness of the given data (no unobserved data). With its high precision, we assume the resulting *X* is the set of all the real causes of *Y*. Furthermore, we select the lagged $Y_{<t}$ from t−k to t−1 as the autoregressive terms, together with *X* to be the set of observed causes;
2. We train the pre-training module and generate $\hat{Y_{t}}$ to estimate $Y_{t}$ ;
3. After finishing training the previous module, we use output $\hat{Y_{t}}$ as the input of this modified NCIE module and train the generator and discriminator (Section 4.1) together to generate *Z*;
4. We train neural network $f_{3}$ (Equation (27)) to generate ${\hat{Y_{t}}}^{\prime }$ , which is applied to examine the improvement of the prediction effect on *Y* given by *Z*.

We note that although there are four neural network architectures in Figure 4 and some of their inputs and outputs are connected, only the two networks in the third module train and update their parameters simultaneously. The framework in Figure 4 proceeds module by module, and the next one starts after the previous one finishes.

#### 4.2.2. Prediction

We test whether the generated *Z* can help to improve the prediction of *Y* by comparing $\hat{Y_{t}}$ and ${\hat{Y_{t}}}^{\prime }$ . In Section 5.2.2, we employ different neural networks to implement $f_{3}$ , which performs the prediction of *Y* by minimizing the mean squared error (MSE):

(27) $${\hat{Y_{t}}}^{\prime }=f_{3}\left(X_{t},\;Y_{t-1},\;Z_{t}\right).$$

## 5. Experiments

### 5.1. Synthetic Data Experiments: Dynamics of Z

**Figure 5:**
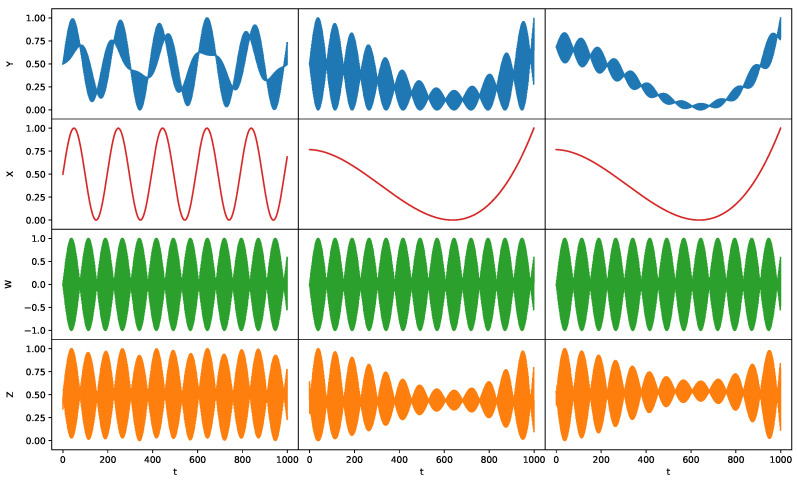
Dynamics of the normalized *Y*, *X*, *W*, and *Z* for Synthetic Case 1 (**Left**), Case 2 (**Middle**), Case 3 (**Right**).

In the following experiments on synthetic data, we demonstrate that the proposed NCIE framework can accurately extract the unobserved causal variables. We consider three synthetic systems (Figure 5) to compare the generated *Z* with the unobserved cause *W* that *Z* mimics. Targets *Y* in the synthetic systems are constructed with causes exhibiting long-term and short-term dynamics. We investigate the ability of NCIE in Section 4.1 to extract causes *W* from target *Y* when *Y* and the other causes *X* are given.

#### 5.1.1. Case 1: A Linear System

We suppose the time-varying *X* and *Y* are observable while *W* is unobserved:

(28) $$\begin{array}{cc} Y(t) & =X(t)+W(t),\\ X(t) & =\sin\frac{t}{10},\\ W(t) & =\sin10t. \end{array}$$

#### 5.1.2. Case 2: A Non-Linear System

We suppose the time-varying *X* and *Y* are observable while *W* is unobserved:

(29) $$\begin{array}{cc} \; & Y(t)=X(t)+W(t)(0.2+X(t)),\\ \; & X\left(t\right)=0.05t^{3}-15t^{2}-80t+2,\\ \; & W(t)=\sin10t. \end{array}$$

#### 5.1.3. Case 3: A Non-Linear System

We suppose the time-varying *X* and *Y* are observable while *W* is unobserved:

(30) $$\begin{array}{cc} \; & Y(t)=X(t)+0.2W(t)(0.2+X(t)),\\ \; & X\left(t\right)=0.05t^{3}-15t^{2}-80t+2,\\ \; & W(t)=\sin10t. \end{array}$$

#### 5.1.4. Case 4: 1—Predator 2—Prey Model Where Prey Share the Same Food

We investigate our approach with the predator–prey model, which is a classical problem in ecology [43,44]. Specifically, we choose the model with 1 predator species, wolves, and 2 prey species, sheep and rabbits. The prey species share the same food source: grass. In total, there are four species in the ecosystem, and their populations grow according to the Lotka–Volterra model [45,46]:

(31) $$\begin{array}{cc} \frac{dW(t)}{dt} & =W\left(t\right)(-a_{0}+a_{1}S\left(t\right)+a_{2}R\left(t\right)),\\ \frac{dS(t)}{dt} & =S\left(t\right)(b_{0}-b_{1}W\left(t\right)+b_{2}G\left(t\right)),\\ \frac{dR(t)}{dt} & =R\left(t\right)(c_{0}-c_{1}W\left(t\right)+c_{2}G\left(t\right)),\\ \frac{dG(t)}{dt} & =G\left(t\right)(d_{0}-d_{1}S\left(t\right)-d_{2}R\left(t\right)), \end{array}$$

where W(t) , S(t) , R(t) , and G(t) represent the population of wolves, sheep, rabbits, and grass at time *t*, respectively. Because this dynamic system can lead to negative populations and we want to prevent extinction from halting our simulation, we add a modification that sets any population to 1 if it drops below 1.

We consider the scenario in which some intermediate species in the biological chain are unknown or unobserved. Therefore, we assume that S(t) and R(t) are unobserved. We set W(t) as the target variable and G(t−1) as the observed cause, and attempt to recover the information provided by the unobserved causes S(t) and R(t) . It is worth noting that G(t−1) is not a direct cause of W(t) in this case. The absence of S(t−1) and R(t−1) makes G(t−1) and W(t) conditionally dependent, thus creating a spurious causal link. Since it is not the focus of our work, we do not discuss how to eliminate spurious links after recovering unobserved causes in this study.

**Figure 6:**
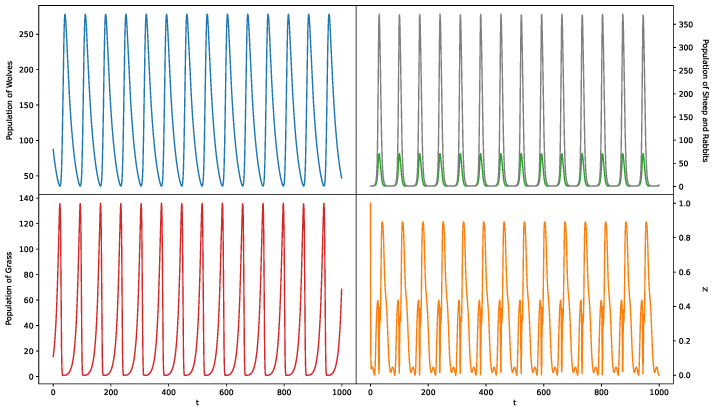
Dynamics of *Z* and the population of four species for Synthetic Case 4.

The dynamics of the population of the four species and *Z* are shown in Figure 6. From this, we can observe that *Z* provides information about the unobserved variables S(t) and R(t) .

#### 5.1.5. The Interpretation of Z

Our target is to generate *Z* that carries the unique information that unobserved causes provide to *Y*. However, in the three synthetic examples, instead of revealing the dynamic of *W*, *Z* exhibits a variable that tries to introduce *Y* while eliminating the information of *X*.

**Figure 7:**
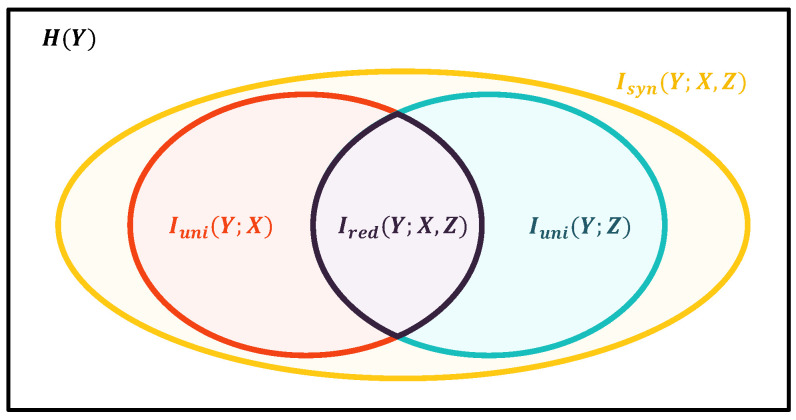
The Venn Diagram illustrates the partial information decomposition of *Y* into four discrete parts: the unique information from *X* (red), the unique information from *Z* (blue), the redundant information from *X* and *Z* (purple), and the synergistic information from *X* and *Z* (yellow).

We try to explain the result using partial information decomposition theory [47], which suggests that we could decompose the Venn Diagram (Figure 2) of mutual information into the following parts shown in Figure 7:

(32) $$\begin{array}{cc} H(Y)=\; & I(Y;X,Y_{<t},W)\\ =\; & I_{\mathrm{uni}}\left(Y;X,Y_{<t}\right)+I_{\mathrm{uni}}\left(Y;W\right)\\ \; & +I_{\mathrm{red}}\left(Y;X,Y_{<t},W\right)+I_{\mathrm{syn}}\left(Y;X,Y_{<t},W\right). \end{array}$$

The information to *Y* from all of its causes consists of four parts:

-
$I_{\mathrm{uni}}\left(Y;W\right)$ , the unique information that could only be provided by *W*;
-
$I_{\mathrm{uni}}\left(Y;X,Y_{<t}\right)$ , the unique information that could only be provided by $\left\{X,Y_{<t}\right\}$ ;
-
$I_{\mathrm{syn}}\left(Y;X,Y_{<t},W\right)$ , the synergistic information provided only when both $\left\{X,Y_{<t}\right\}$ and *W* are present;
-
$I_{\mathrm{red}}\left(Y;X,Y_{<t},W\right)$ , the redundant information that could be provided by either $\left\{X,Y_{<t}\right\}$ or *W*.

If our objective is to recover *W*, *Z* should only contain the unique information $I_{\mathrm{uni}}\left(Y;W\right)$ and the synergistic information $I_{\mathrm{syn}}\left(Y;X,Y_{<t},W\right)$ , while the unique information $I_{\mathrm{uni}}\left(Y;X,Y_{<t}\right)$ and the redundant information $I_{\mathrm{red}}\left(Y;X,Y_{<t},W\right)$ are not present in this case. However, our objective in generating *Z* is not to exactly mimic *W*, but to maximize the information that the observed and unobserved causes provide to *Y*, so that we can acquire a better causal representation for *Y*.

Moreover, it is difficult to integrate different methods; therefore, our architecture does not measure the four components [48,49,50] separately but rather measures and maximizes the entire $I(Y;X,Y_{<t},Z)$ at once. Thus, $I_{\mathrm{uni}}\left(Y;Z\right)$ , $I_{\mathrm{syn}}\left(Y;X,Y_{<t},Z\right)$ , and $I_{\mathrm{red}}\left(Y;X,Y_{<t},Z\right)$ in Equation (32) are maximized. As a result, the implicit causes *Z* may contain all four components, which is the information from both the observed $\left\{X,Y_{<t}\right\}$ and the unobserved *W*.

Therefore, in the synthetic cases, we still observe the trend information from *X* that is not fully eliminated in *Z*. Still, in this case, we argue that *Z* carries significant information and reveals evident dynamics of *W*. As shown in Table 1, the observed causes *X* only provide very little information to the target *Y* in simple synthetic cases, where the mutual information of target *Y* provided by itself, I(Y;Y) , is equivalent to the entropy of *Y*, H(Y) , provided that we consider *Y* as a random variable. In contrast, after we add the implicit causes *Z*, all the causes together provide most of the information of target *Y* and thus complete the causal structure of *Y*.

### 5.2. Preformance Evaluation by Real-World Data Experiments

#### 5.2.1. Information Recovery

We first examine the ability of NCIE (Section 4.1) to extract implicit causes that recover unobserved information of the targets in several real-world datasets, including

- The Electricity Transformer (Oil) Temperature (ETT) [17], which includes four datasets sampled at different time intervals (1 min, 2 min, 1 h, and 2 h). The observed causes include useful and useless loads in different levels;
- The daily exchange rates [51] of eight countries, with one of them set as the target and the other seven as the observed causes for predicting the target;
- Minneapolis–St Paul interstate metro traffic volume [52], in which observed causes include temperature, weather, and holidays;
- The PM2.5 quantity in Beijing (https://www.kaggle.com/datasets/rupakroy/lstm-datasets-multivariate-univariate) (accessed on 6 November 2023), in which observed causes include dew, temperature, atmospheric pressure, weather, as well as wind direction and speed (all the codes and data are available at https://github.com/jh-liang/NeuralCausalInfoExtractor) (accessed on 6 November 2023).

These data provide partial observations of complete systems in industrial monitoring, financial markets, and urban operations. In the provided datasets, it is obvious that some causes, *W*, are unobserved. For example, exchange rates are influenced not only by foreign exchange rates but also by the domestic economy and complex international trade. Similarly, the PM2.5 datasets do not involve urban human activities and pollution emissions from power plants. We aim to recover conditional information I(W;Y|X) using *Z*, which advances the precision of time series forecasting.

Table 2 shows the information provided by only observed causes and by the combination of observed and implicit causes. It illustrates that the generated *Z* can provide significant extra information besides the observed causes *X* in real-world scenarios. The *Z* in this table is generated by NCIE (Section 4.1). We do not discuss the dimensionality of *Z* in this paper. While the higher dimensionality of *Z* provides the wider information channel, we simply set the dimension of *Z* to be the dimension of $\left\{X,Y_{<t}\right\}$ .

#### 5.2.2. Single-Step Time Series Forecasting

While we claim that our architecture helps to identify unobserved causes of the target variables, it holds massive potential for extracting useful information from the available features and for conducting time series forecasting. We evaluate our architecture by performing time series forecasting on the real-time series datasets and comparing our results with the baselines [18,19] listed in Table 3. To prevent future information leakage in time series prediction, we employ the modified NCIE architecture in Section 4.2 to generate *Z*.

In this work, we are considering one-dimensional targets *Y* with multiple features that are candidates for observed causes chosen by PCMCI. Additionally, our architecture focuses on single-step time series forecasting because our goal is to find the direct causes for *Y*, which could be $X_{t-k}$ and $Z_{t-k}$ with a small lag *k*. The results are shown in Table 3. For the three chosen time series analyzing tools, we compare the prediction results with and without *Z*. It is illustrated that including *Z* in most cases does outperform those predictions without implicit variables *Z*.

Our problem could be considered a feature selection task. The *Z* we generate could be considered as a feature that helps to describe *Y*. We see that in a few cases, *Z* does not necessarily improve the prediction of *Y*. This could be due to two reasons. First, although we know there is a gap between the entropy of *Y* and the information that *X* and $Y_{<t}$ bring to *Y*, this gap might be too narrow for NCIE to generate a *Z* that brings sufficient information from it. Second, when the system is time-varying, the underlying causality structure of the system could also vary with time. *Z* that acts as a significant cause in (part of) the training dataset may become a redundant variable that perturbs the prediction.

It is worth mentioning that RNN and LSTM do not outperform simple neural networks (NN) in all cases. Our explanation is that the features *X* that PCMCI chooses cover most of the (observed) direct causes of *Y*, and we also choose autoregressive features $Y_{<t}$ from t−k to t−1 that already cover short- and long-term memories of *Y*. Therefore, the memories that RNN and LSTM extract could not significantly help the prediction, but, in contrast, overfit *Y*.

Table 4 shows the mutual information between different sets of variables and *Y*. For real data, the *Z* that NCIE generates can provide extra information to *Y*. It demonstrates NCIE’s ability to extract part of the implicit causes in the system, although we find a gap between $I(Y;X,Y_{<t},Z)$ and I(Y;Y) . It is unavoidable in cases in which the systems are open or noisy because external noises are not driven by variables within the system. Furthermore, for a large system with too many causes, some causes may carry very little unique information to the target, and it is difficult to express them with limited *Z*.

The first column of Table 4 shows the mutual information between the pre-trained $\hat{Y}$ and the actual *Y*. We recall that the pre-trained $\hat{Y}$ is generated by an RNN architecture that minimizes the mean square error (MSE), given the explicit causes *X* and $Y_{<t}$ . The gap from the first column to the others shows that RNN and other recurrent structures do not necessarily provide a regression result with sufficient information to the target. While it is not a key point to argue the effectiveness of different objectives in this work, it reminds us that NCIE provides more information than general neural architectures.

## 6. Conclusions

In this work, we propose NCIE to generate implicit variables that complement the unobserved information of the target not provided by the observed variables. We can complete the causal structure of the target with the implicit variables while retaining the observed causes, as they together provide most of the target’s information. Furthermore, the generated implicit variables have similar dynamics to the unobserved causes in the synthetic experiments and help to predict the target in the real-world time series. These results provide sufficient evidence that the implicit variables can be an effective candidate to substitute the unobserved causes and complete the causal structure.

While this work only focuses on discovering unobserved causes for single targets, future attempts could be made to apply similar methods to construct a complete graph for multiple targets, in which all the causes provide complete information for all the targets. With such methods, we are able to depict the evolution of systems by the information flow among variables, which could contribute to the research of emergence in real-world complex systems.

## Figures and Tables

**Table 1:** Mutual information provided to *Y* from different sets of variables.

Case	Explicit *X*	Explicit *X* + Implicit *Z*	Target *Y*
1	0.646	5.46	5.52
2	0.679	4.11	5.35
3	1.44	3.47	5.29
4	2.84	3.48	5.36

**Table 2:** Mutual information provided to *Y* from different sets of variables. The *Z* in this table is generated by the original NCIE (Section 4.1).

Datasets	Explicit	Explicit + Implicit *Z*	Target *Y*
ETTh1	2.78	5.54	6.75
ETTh2	3.55	5.55	6.66
ETTm1	3.12	5.46	6.33
ETTm2	4.278	5.36	6.27
ExcRat	3.63	6.24	7.31
Metro	2.66	5.68	7.37
PM2.5	1.80	4.59	5.52

**Table 3:** Single-step time series forecasting with and without *Z* in three different architectures, where ’NN’ denotes the result from a simple NN with input including only observed causes, and ’I-NN’ denotes the result with input including both observed causes and implicit variables *Z* generated by modified NCIE. The better result in the comparison is underlined.

Datasets	NN	I-NN	RNN	I-RNN	LSTM	I-LSTM
ETTh1	1.97 × 10 ${\;}^{-4}$	1.89 × 10 ${\;}^{-4}$	1.65 × 10 ${\;}^{-4}$	1.77 × 10 ${\;}^{-4}$	2.05 × 10 ${\;}^{-4}$	2.01 × 10 ${\;}^{-4}$
ETTh2	1.81 × 10 ${\;}^{-4}$	1.45 × 10 ${\;}^{-4}$	2.05 × 10 ${\;}^{-4}$	1.28 × 10 ${\;}^{-4}$	2.62 × 10 ${\;}^{-4}$	1.64 × 10 ${\;}^{-4}$
ETTm1	4.19 × 10 ${\;}^{-5}$	4.16 × 10 ${\;}^{-5}$	4.35 × 10 ${\;}^{-5}$	4.24 × 10 ${\;}^{-5}$	7.57 × 10 ${\;}^{-5}$	5.12 × 10 ${\;}^{-5}$
ETTm2	2.22 × 10 ${\;}^{-5}$	1.68 × 10 ${\;}^{-5}$	4.59 × 10 ${\;}^{-5}$	3.37 × 10 ${\;}^{-5}$	1.45 × 10 ${\;}^{-4}$	8.17 × 10 ${\;}^{-5}$
ExcRat	9.90 × 10 ${\;}^{-5}$	9.84 × 10 ${\;}^{-5}$	2.01 × 10 ${\;}^{-4}$	1.99 × 10 ${\;}^{-4}$	4.25 × 10 ${\;}^{-4}$	3.57 × 10 ${\;}^{-4}$
Metro	3.33 × 10 ${\;}^{-3}$	2.91 × 10 ${\;}^{-3}$	4.09 × 10 ${\;}^{-3}$	4.03 × 10 ${\;}^{-3}$	4.36 × 10 ${\;}^{-3}$	3.94 × 10 ${\;}^{-3}$
PM2.5	5.50 × 10 ${\;}^{-4}$	5.46 × 10 ${\;}^{-4}$	5.87 × 10 ${\;}^{-4}$	5.78 × 10 ${\;}^{-4}$	5.87 × 10 ${\;}^{-4}$	5.70 × 10 ${\;}^{-4}$

**Table 4:** Mutual information provided to *Y* from different sets of variables. The *Z* in this table is generated by the modified NCIE (Section 4.2).

Datasets	Pre-Trained $\hat{Y}$	Explicit	Explicit + *Z*	*Y*
ETTh1	1.46	3.59	3.60	6.31
ETTh2	2.56	3.65	3.90	6.69
ETTm1	2.51	3.05	2.94	5.58
ETTm2	3.39	4.36	4.37	6.23
ExcRat	2.87	2.98	4.14	6.88
Metro	1.06	3.42	3.45	7.60
PM2.5	0.96	2.32	2.34	6.53

## Data Availability

All the codes and data are available at https://github.com/jh-liang/NeuralCausalInfoExtractor (accessed on 6 November 2023).
